# Electrochemically tuneable multi-colour electrochemiluminescence using a single emitter†
†Electronic supplementary information (ESI) available. See DOI: 10.1039/c6sc01912a



**DOI:** 10.1039/c6sc01912a

**Published:** 2016-07-22

**Authors:** Mohammad A. Haghighatbin, Shih-Chun Lo, Paul L. Burn, Conor F. Hogan

**Affiliations:** a Department of Chemistry and Physics , La Trobe Institute for Molecular Sciences , La Trobe University , Melbourne , Victoria 3086 , Australia . Email: c.hogan@latrobe.edu.au; b Centre for Organic Photonics & Electronics (COPE) , The University of Queensland , School of Chemistry and Molecular Biosciences , Brisbane , Queensland 4072 , Australia

## Abstract

A single starting component electrochemiluminescence system from which red, green, blue or white emission can be obtained, depending on the applied potential or the mode of the ECL experiment, is described. The convoluted ECL spectral responses observed at different potentials are readily explained using a 3D-ECL technique, where the ECL spectral profile is continuously monitored as a function of potential during voltammetric scanning. The 3D plots obtained using this technique implicate cross-annihilation ECL reactions involving the complex itself and stable products resulting from its electrolysis. Combining this information with knowledge of the energetic requirements of the various reactions involved, suggests a mechanism involving traces of two emissive products, related to the loss of a methyl group from the triazole moiety. These products, while barely detectable electrochemically, are sufficiently emissive to influence and even dominate the ECL emission under some conditions.

## Abstract 2

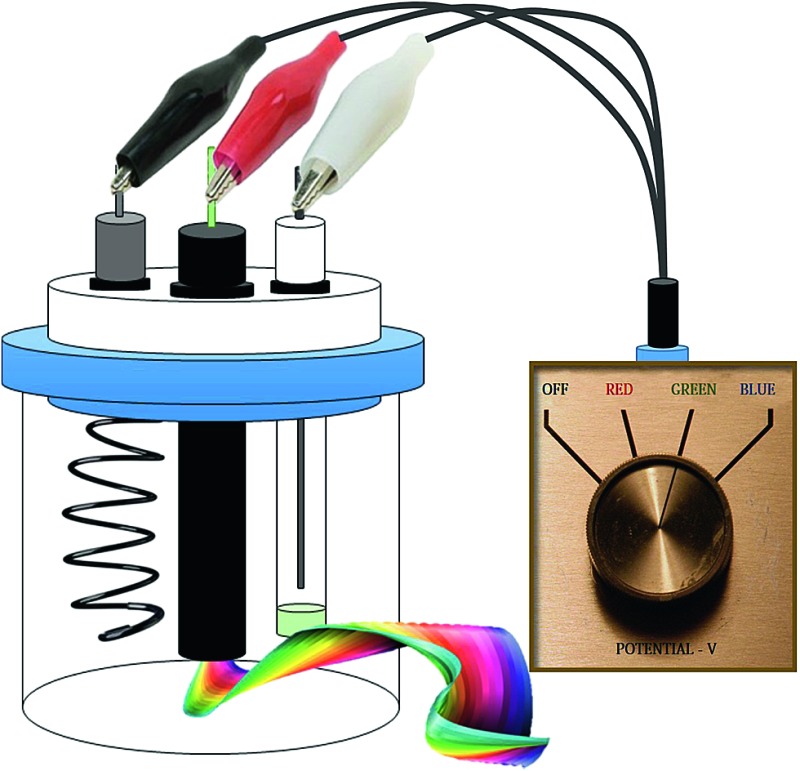
A single component electrochemiluminescence system from which red, green, blue or white emission can be obtained, depending on the applied potential or the mode of the ECL experiment, is described.

## Introduction

The electrochemical generation of emissive excited states known as electrochemiluminescence or electrogenerated chemiluminescence (ECL) continues to grow in importance as an area of research.^1–3^ This is principally due to the utility of the phenomenon as the basis for a wide range of highly sensitive analytical and bioanalytical techniques,^4,5^ but also due to its potential for the development of light emitting devices (LEDs) based on emissive molecular species. One aspect of ECL which is currently attracting attention is the possibility of generating and detecting multiple emissions simultaneously or sequentially in a single system. Such multi-colour ECL systems are attractive because they open the possibility of multiplexed ECL analysis, *i.e*., analysis of multiple electrochemiluminophores without separation; and suggest a new approach to colour tuneable light emitting devices.^6^ Detection of more than one ECL emitter was first reported by Richter^7,8^ who observed simultaneous ECL from a mixture of red-emitting [Ru(bpy)_3_]^2+^ and green emitting *fac*-[Ir(ppy)_3_], in the presence of tri-*n*-propylamine (TPrA) as a co-reactant, while applying a single potential. Later, Forster and co-workers^9^ used an electrode modified with an ECL-active bimetallic polymer containing ruthenium and osmium polypyridyl centres, emitting at 600 and 760 nm, respectively, for selective determination of guanine and oxoguanine as co-reactants. Hogan, Francis and co-workers first showed the possibility of selectively exciting co-reactant ECL from a mixture of two or more luminophores with a distinct difference between their emission colours and/or oxidation potentials.^10–12^ More recently, the ability to modulate the colour of annihilation ECL emission in mixtures of luminophores was also demonstrated by these authors.^13^

Apart from these so-called mixed ECL systems, variable-colour ECL from a single molecular emitter has been only occasionally reported; usually resulting from either formation of an emissive excimer^14–17^ or from emissive products of chemical or electrochemical side reactions.^18^ For example, Bard and co-workers^19^ recently reported two-colour ECL from BODIPY-appended 2,2′-bipyridine homologues (where BODIPY is 4,4-difluoro-4-boro-3*a*,4*a*-diaza-*s*-indacene) during annihilation ECL experiments. The bifurcated ECL was ascribed to emission from BODIPY at 570 nm and a possible dimerisation or oligomerisation product of the electrochemical oxidation process at 740 nm. Ding *et al.* recently described a hybrid system comprising PbS quantum dots capped with BODIPY which emitted concurrently in the red and near infrared on electrochemical excitation.^20^ Zysman-Colman and co-workers^21,22^ in another example observed a significant red-shift (535 to 610 nm) in the ECL spectrum of an iridium complex containing a dimethyl-amino bipyridine ligand, when a sufficiently high potential was applied such that the amine moiety was irreversibly oxidised, thus affecting a change in its electron withdrawing/donating character. Schmittel and co-workers^23^ also reported a potential dependent ECL emission from a trinuclear Ir(iii)–Ru(ii)–Ir(iii) system. Here, a small step-less hypsochromic shift in the ECL emission was observed in the range 611–649 nm due to variations in the contributions of the frontier orbitals to the excited state with increasing oxidation state.

However, despite the interesting nature of these various reports, a single-luminophore system where the emission colour can be readily modulated by simply varying the applied potential, as demonstrated for mixed ECL systems, has not been reported. Moreover, the lack of a convenient method of probing the multi-colour ECL in such unusual systems has resulted in a dearth of mechanistic investigations in multi-colour ECL systems. We recently introduced a new approach to the investigation of multi-component ECL systems using a technique referred to as 3D-ECL.^10–12,24^ Conceptually similar to excitation–emission matrix experiments common in studies of photoluminescent systems, collected ECL spectra as a function of applied potential during a voltammetric scan or series of steps. The chief benefit of this method is that it is possible to capture the totality of information relating to a mixed ECL system in a single three-dimensional graph of ECL intensity *versus* potential and wavelength. While these studies focused on multi-component systems with a view to possible applications in multiplex ECL analysis, the technique has not previously been used for mechanistic investigation of a multi-colour ECL system based on a single starting component.

In this contribution we demonstrate an unusual ECL behaviour of *fac*-tris[5-(4-fluoro-3-methylphenyl)-1-methyl-3-*n*-propyl-[1,2,4]-triazolyl]iridium(iii) complex, Ir(mptz)_3_, a complex previously investigated for applications in OLEDs.^25^ We show that several different emission colours, including near white, can be reversibly generated by changing the applied potential. We also investigate the annihilation ECL mechanism using the 3D-ECL technique to obtain information not readily obtainable by traditional methods.

## Results and discussion

**Fig. 1:**
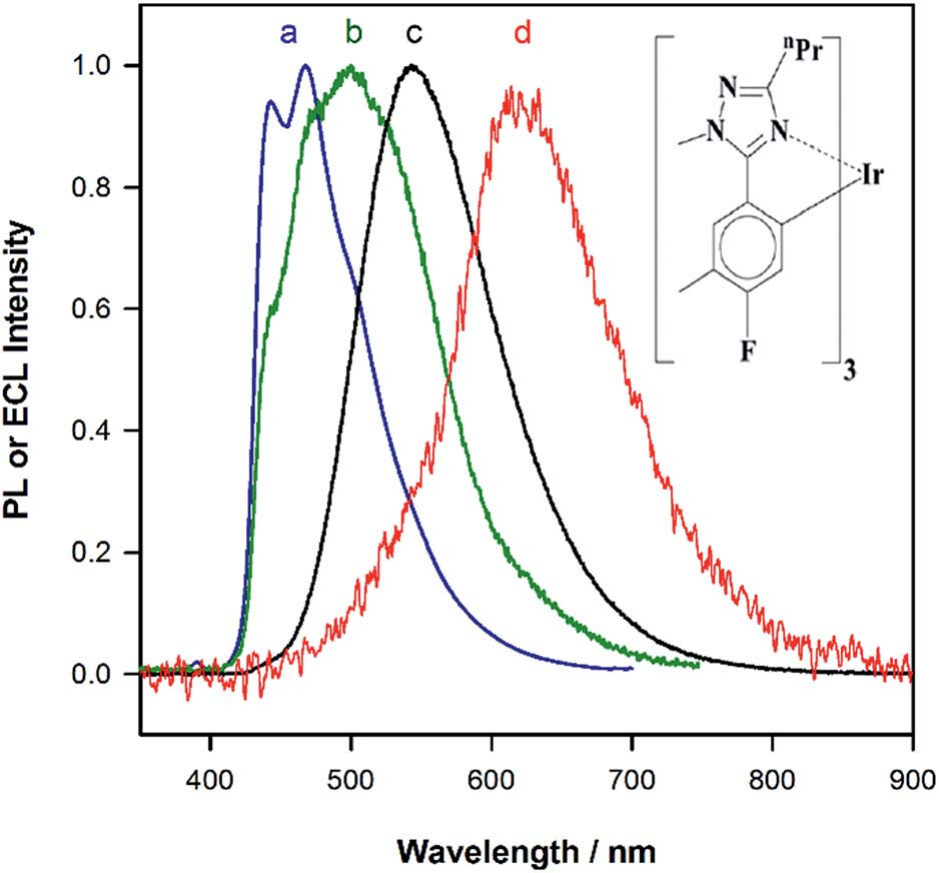
(a) Photoluminescence spectrum of a 0.01 mM solution of Ir(mptz)_3_ in degassed acetonitrile (298 K), (excitation wavelength: 350 nm). (b–d) Potential dependent electrochemiluminescence (ECL) spectra of a 0.5 mM solution of Ir(mptz)_3_ in acetonitrile/0.1 M TBAPF_6_, collected by pulsing the potential between 0.49 V and (b) –3.20 V, (c) –2.56 V, (d) –2.36 V *versus* Fc^+^/Fc. Intensities normalised. The chemical structure of Ir(mptz)_3_ is shown in the inset.

Fig. 1a shows the photoluminescence (PL) spectrum of Ir(mptz)_3_ in degassed acetonitrile solution. The blue phosphorescence observed under these conditions has a structured emission profile, with peaks at 433 nm and 469 nm, suggesting that the nature of the excited state is a mixture of ^3^LC (ligand centred) and ^3^MLCT (metal-to-ligand charge transfer).^26^ Annihilation ECL experiments were carried out by pulsing the working electrode potential between a value more positive than the oxidation potential for the complex [*E*oox = 0.39 V *versus* ferrocenium/ferrocene (Fc^+^/Fc)] and more negative than its first reduction potential (*E*ored = –3.14 V *versus* Fc^+^/Fc). In the vast majority of reported ECL systems, the ECL spectral profile matches that of its photoluminescence (PL). Unexpectedly, however, in this case the ECL spectrum, as shown in Fig. 1b, exhibits a quite dissimilar emission profile; the peak is considerably broader and its maximum is bathochromically shifted by ∼40 nm. It is also clear from Fig. 1b, however, that the ECL profile contains features similar to the PL spectrum, suggesting that two apparently convolved emissions are involved. Interestingly, when the potential step sequence used to generate annihilation ECL was made less negative than the first reduction process, (*i.e.*, here –2.56 V *versus* Fc^+^/Fc), the ECL spectrum was further red shifted resulting in very intense yellow-green emission centred at 550 nm (Fig. 1c). Finally, for even less cathodic limits around –2.36 V *versus* Fc^+^/Fc, a weaker red emission with a maximum at 609 nm was observed, (see Fig. 1d above and S1 in ESI†).

**Fig. 2:**
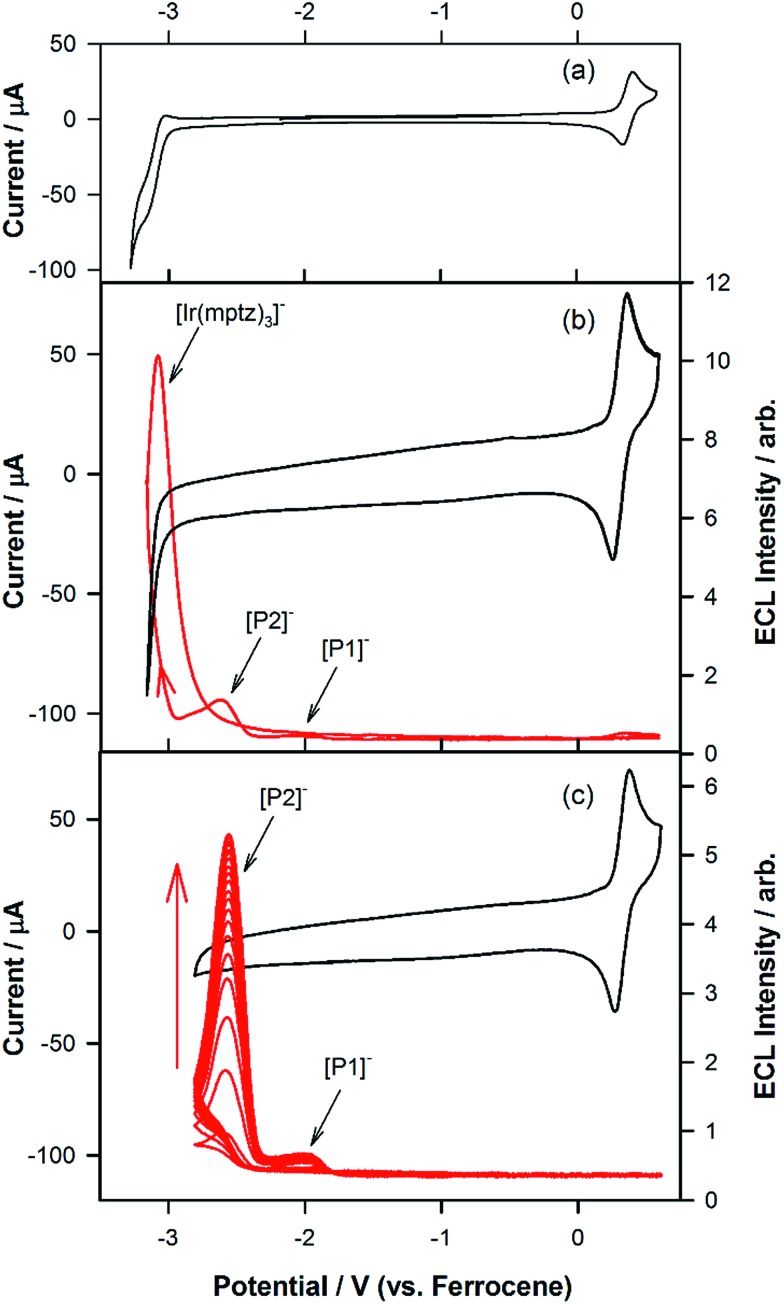
(a) Cyclic voltammogram at a scan rate of 0.1 V s^–1^ for 0.5 mM Ir(mptz)_3_ in acetonitrile containing 0.1 M TBAPF_6_. (b) Cyclic voltammogram in the range of 0.6 V and –3.2 V *versus* Fc^+^/Fc at 0.1 V s^–1^ overlaid with the ECL signal detected using a PMT. (c) Cyclic voltammogram overlaid with the ECL signal in the range of 0.6 V and –2.8 V *versus* Fc^+^/Fc at a scan rate of 1.0 V s^–1^.

To investigate the origin of the unusual ECL spectral profiles in more detail, we tracked the ECL signal as a function of potential using a photomultiplier tube (PMT) as the detector. Fig. 2a shows the cyclic voltammogram of Ir(mptz)_3_ in acetonitrile. Fig. 2b shows the PMT detector response during an oxidative–reductive annihilation ECL experiment where, in order to generate the oxidised and reduced annihilation partners, the potential was first scanned 50 mV past the anodic peak, then to the negative limit. To avoid potential interference from the solvent electrolysis products, the potential was swept no further than the rising portion of the cathodic peak during the experiment shown in Fig. 2b.

Following the initial anodic sweep, on the negative-going scan, three distinct peaks in the ECL signal were observed at –1.95, –2.56 and –3.14 V *versus* Fc^+^/Fc. The peak at –1.95 V was somewhat lower in magnitude than the other two but could be enhanced by increasing the scan rate or by continuous cycling to the anodic limit and back, (see Fig. 2c). Importantly, no emission was observed unless the potential was initially swept into the anodic region of the voltammogram, where [Ir(mptz)_3_]^+^ is generated, see Fig. S2 in the ESI.† While the ECL peak at –3.14 V can readily be assigned to the annihilation reaction between [Ir(mptz)_3_]^+^ and [Ir(mptz)_3_]^–^, which is generated at this potential (see eqn (1) below), the lack of any obvious current peaks in the voltammogram corresponding to the ECL signals at –1.95 V and –2.56 V seems anomalous. The most likely explanation for this is the formation of two strongly emissive products, designated here as P1 and P2, in very low concentrations during the oxidative scan, with reduction potentials close to –1.95 V and –2.56 V, respectively. Note that the apparent lack of faradaic current signal corresponding to their reduction can be understood by bearing in mind the significant difference in sensitivity between the electrochemical and photonic modes of detection (detection limits for ECL can be up to six orders of magnitude lower).^27^ To test this hypothesis, the potential was continuously cycled between the oxidation peak and –2.80 V. The results shown in Fig. 2c appear to bear the theory out; with the ECL signals at –2.56 and –1.95 V growing significantly with scan number, consistent with the build-up of new ECL-active products, P1 and P2, in the diffusion layer. Simulations carried out using KISSA^28^ confirm that sufficient quantities of oxidation products to give rise to a large ECL signal can be generated without either the appearance of detectable reduction currents or a noticeable effect on the chemical reversibility of the oxidation process.

The chemical and electrochemical processes leading to the unusual ECL behaviour of this system may be summarised as follows:

1 [Ir(mptz)_3_] ↔ [Ir(mptz)_3_]^+^ + e^–^

2a [Ir(mptz)_3_]^+^ → [P1]^+^

2b [Ir(mptz)_3_]^+^ → [P2]^+^

3a [P1] + e^–^ ↔ [P1]^–^ (at –1.95 V)

3b [P2] + e^–^ ↔ [P2]^–^ (at –2.56 V)

3c [Ir(mptz)_3_] + e^–^ ↔ [Ir(mptz)_3_]^–^ (at –3.14 V)

4a [P1]^+^ + [P1]^–^ → [P1] + [P1]*

4b [P2]^+^ + [P2]^–^ → [P2] + [P2]*

4c [Ir(mptz)_3_]^+^ + [Ir(mptz)_3_]^–^ → [Ir(mptz)_3_] + [Ir(mptz)_3_]*

5a [P1]* → [P1] + *hν* (∼600 nm)

5b [P2]* → [P2] + *hν* (∼550 nm)

5c [Ir(mptz)_3_]* → [Ir(mptz)_3_] + *hν* (∼448 nm)

Preliminary work to elucidate the structure of these electrochemical products (see Fig. S6a and S6b in ESI†), suggest that they result from ligand-based reactions, related to the loss of the methyl group attached to the nitrogen of the triazole moiety. Dissociation of the methyl group from the triazole ring would have the effect of stabilising the LUMO, resulting in a red-shifted ECL signal and a less negative reduction potential, both of which are consistent with experimental observations. The very low annihilation ECL signal intensity at the anodic end of the voltammogram (combined with the chemically reversible oxidation process in the cyclic voltammogram) is noteworthy, suggesting that the oxidised complex and the oxidatively generated products are more stable than the corresponding reduced forms.

**Fig. 3:**
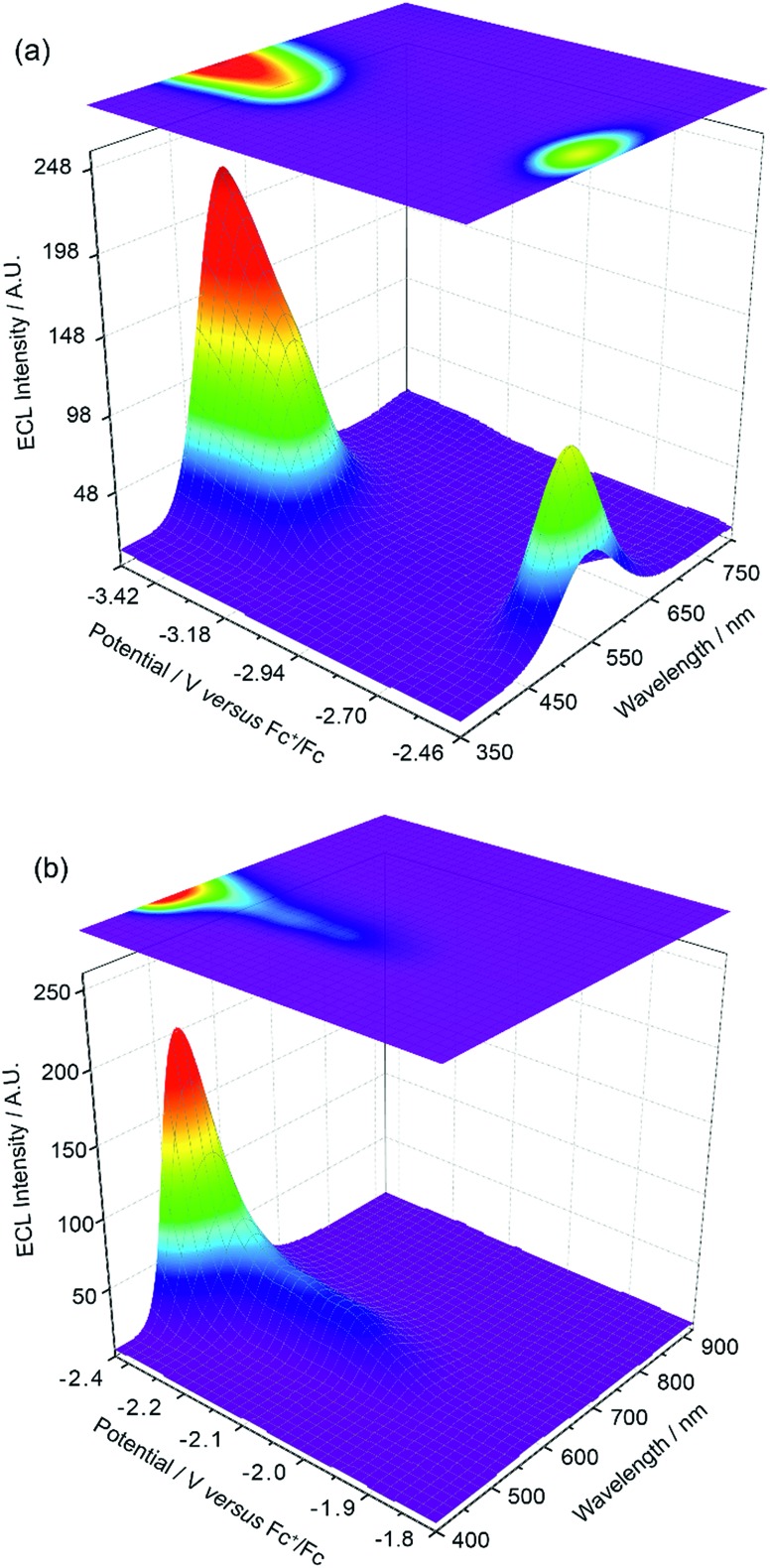
(a) 3D-ECL plot for 0.5 mM Ir(mptz)_3_ in acetonitrile/0.1 M TBAPF_6_, generated using a 0.05 V s^–1^ voltammetric scan in the range of –2.46 V and –3.51 V *versus* Fc^+^/Fc. (b) 3D-ECL plot generated using chronoamperometry by pulsing for 0.25 s between 0.49 V and –1.81 to –2.46 *versus* Fc^+^/Fc with the increments of 0.05 V.

The experiments represented in Fig. 1 and 2 in isolation offer important though limited insight into the reasons for the unusual ECL behaviour observed in this system. Therefore, we carried out further investigations using a relatively new methodology referred to as 3D-ECL^10,11^ where the ECL spectral profile is measured as a function of scanned potential. To perform the experiment shown in Fig. 3a, the solution was pre-electrolysed for 10 seconds at 0.49 V *versus* Fc^+^/Fc and then a reductive voltammetric scan immediately performed from –2.46 V to –3.51 V at a scan rate of 0.05 V s^–1^, (see Fig. S3† for details of the excitation signal). Due to the relatively lower intensity of the red ECL emission process at –1.95 V, this region (Fig. 3b), was run as a separate experiment using incremental potential pulses, (see Experimental section) rather than linear scan voltammetry, in order to achieve higher sensitivity.

Fig. 3a and b expose subtleties in the ECL behaviour of the system not revealed by the previous experiments. For example, the emission process at –3.14 V can now be readily seen to be characterised by a gradual transition from a green dominated emission to two distinct components on scanning from –3.05 V to –3.35 V. Moreover, the process at –2.56 V, while dominated by green emission appears to have a red component at lower potentials and a slight blue component at higher potentials. Finally, the ECL emission peak at –1.95 V in Fig. 3b can be seen to be composed of a single component with *λ*_max_ = 609 nm, up to –2.35 V, before gradually transitioning into the green emission at –2.56 V.

An inescapable conclusion from Fig. 3 is that the mechanism outlined in eqn (1–5) above is insufficient to explain all the features of the 3D graph. In particular, the existence of a blue component to the emission at –2.56 V, prior to the generation of [Ir(mptz)_3_]^–^, seems anomalous. This suggests that cross annihilation reactions, *i.e.*, ECL resulting from annihilation between partners of differing identity, need to be considered. For example, the red emission observed at –1.95 V may be produced not only by reaction 4a above, but by annihilation between [Ir(mptz)_3_]^+^ and [P1]^–^.

Table 1 summarises all possible cross reactions involving [Ir(mptz)_3_]^+^. This table predicts the emission colours possible for each potential on the basis of thermodynamics by considering the following criterion for an energy sufficient ECL reaction:^29^
6
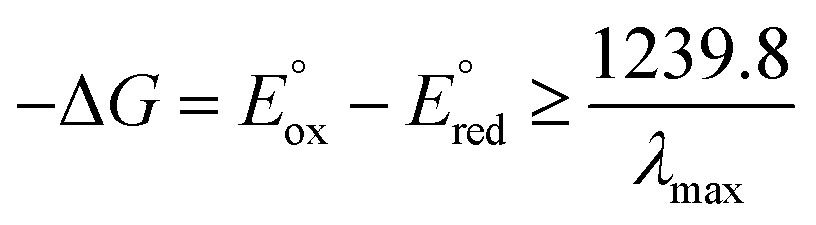
where *E*oox and *E*ored for Ir(mptz)_3_ is 0.39 V and –3.14 V *versus* Fc^+^/Fc, respectively, the values of *E*ored for P1 and P2 are approximated by the positions of the ECL maxima in Fig. 2, and the *λ*_max_ for the ECL emissions (460, 550 and 609 nm) approximate the energies of the excited state products. Table 1 well explains the colours observed at each potential; predicting only the red emission at –1.95 V and a convolved emission from P1, P2 and Ir(mptz)_3_ at more negative potentials. In particular it shows how blue emission can be observed at –2.56 V without generating [Ir(mptz)_3_]^–^.

**Table 1:** Analysis of possible outcomes of ECL annihilation cross reactions between oxidised and reduced forms of the complex and its two oxidation products (P1 and P2), based on evaluation of their energy sufficiency using eqn (6)

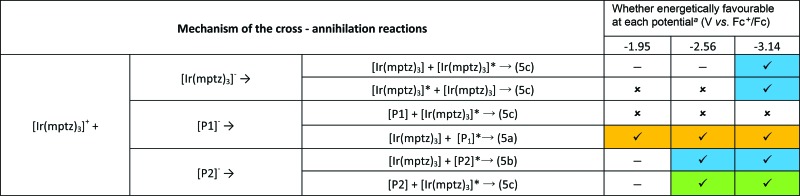

**Fig. 4:**
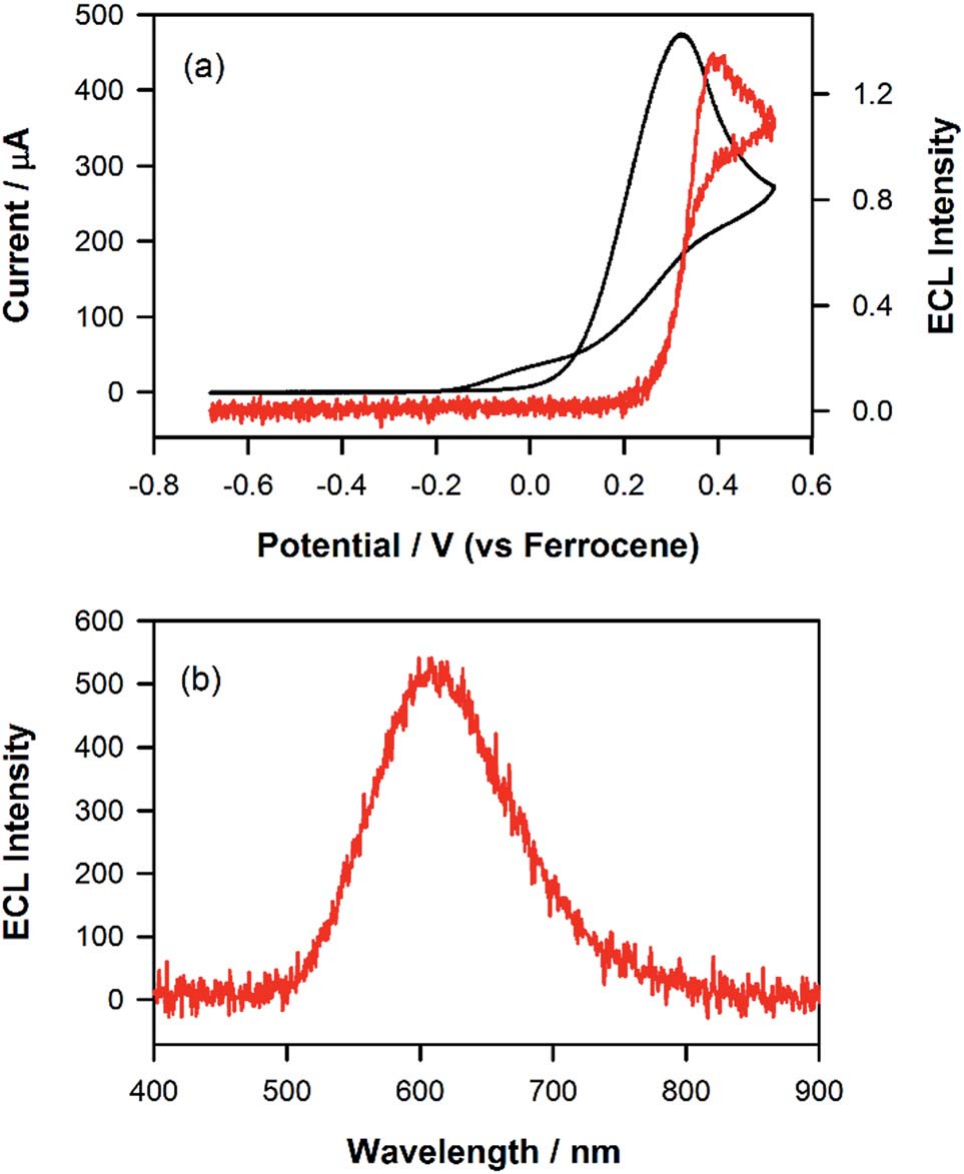
(a) Cyclic voltammogram of 0.5 mM Ir(mptz)_3_ in acetonitrile containing 0.1 M TBAPF_6_ as supporting electrolyte and 1 mM TPrA as co-reactant. The red trace shows the concurrent ECL signal (PMT output) (scan rate: 0.1 V s^–1^). (b) Co-reactant ECL spectrum of the same solution generated by pulsing the potential between 0.0 and 0.5 V *versus* Fc^+^/Fc with a pulse width of 0.25 s for 12 cycles. (CCD integration time: 6 s).

One way to further test the hypothesis outlined above would be to attempt to generate co-reactant ECL using tri-*n*-propylamine (TPrA). As this co-reactant forms a reductant with *E*ored = –2.1 *versus* Fc^+^/Fc, on oxidation,^30^ population of either the blue or green excited state (*i.e*., [Ir(mptz)_3_]* and [P2]*) ought to be precluded thermodynamically. As shown in Fig. 4, when such an experiment was performed using a 0.5 mM acetonitrile solution of the complex in the presence of 1 mM TPrA, emission centred at 609 nm consistent with that when only the formation of [P1]* was observed. Analogous to the situation with the annihilation reaction at –1.95 V described above, this demonstrates that the ECL due to [P1]^+^ is selectively generated by the co-reactant pathway due to the energy insufficiency of the corresponding reactions involving [P2]^+^ and [Ir(mptz)_3_]^+^.

**Fig. 5:**
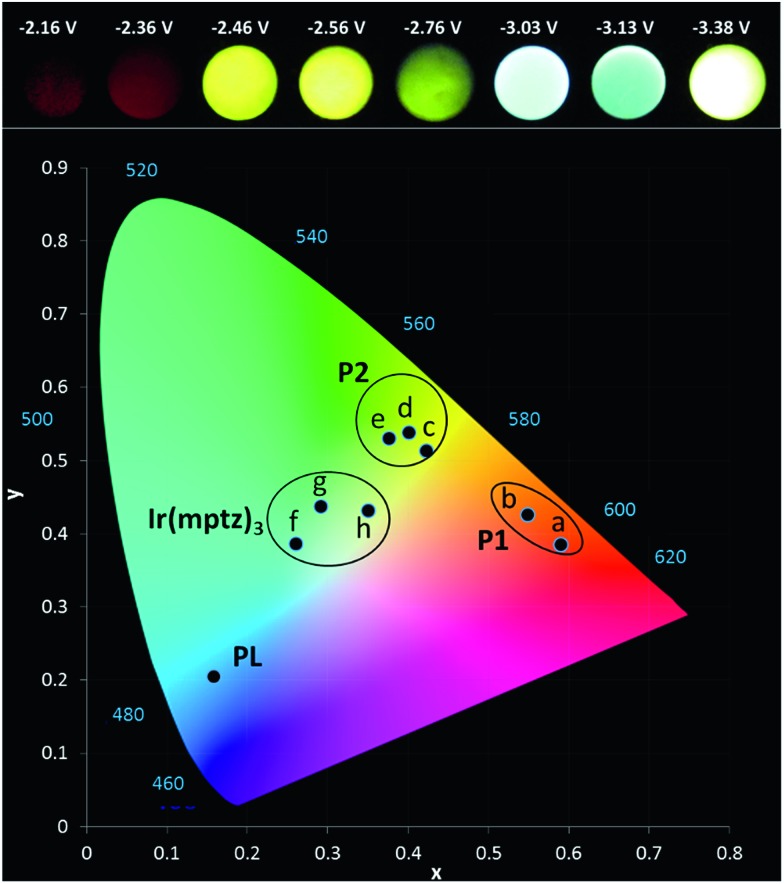
(a) Photographs of the ECL at a glassy carbon electrode (3 mm) (b) CIE 1931 (2-degree observer) chromaticity diagram of the ECL spectra. Conditions: 0.5 mM Ir(mptz)_3_ and 0.1 M TBAPF_6_ in acetonitrile pulsed between 0.49 V and (a) –2.16, (b) –2.36, (c) –2.46, (d) –2.56, (e) –2.76, (f) –3.03, (g) –3.13, (h) –3.38 V *versus* Fc^+^/Fc. Solution purged with N_2_ and kept under a N_2_-blanket during the experiments. 15 s exposure time considered for each photograph.

Owing to the convoluted nature of the ECL spectra in Fig. 1 and in the 3D-ECL graphs, the ECL *λ*_max_ is a poor indication of the actual emission colour produced. Fig. 5 shows a series of photos taken from the surface of the electrode during a reductive sweep of the potentials. A range of colours, from orange to green-blue, were observed for the annihilation ECL. Significantly, near-white emission was observed at potentials close to –3.3 V *versus* Fc^+^/Fc which is a result of a simultaneous emission from the complex and the two products, (see Fig. S5†). Due to interest in potential tuneable OLEDs and LEECs, it is important to elucidate the actual colour of the ECL at different potentials. Therefore, we generated the 1931 CIE (Commission internationale de l'éclairage) chromaticity coordinates (2-degree observer) for different reductive potentials. Fig. 5 and S4† show the CIE coordinates of the ECL emission obtained by varying the reduction potential from –2.16 to –3.38 V *versus* Fc^+^/Fc. Three distinct clusters related to P1 (orange), P2 (yellow-green) and Ir(mptz)_3_ (green-blue) were observed depending on reduction potentials. At potentials of <–2.36 V *versus* Fc^+^/Fc, emission at 609 nm is the only emission observed in the orange zone of the CIE diagram with the coordinates of (0.55, 0.43), whereas, at potentials of –2.6 V *versus* Fc^+^/Fc the emission is localised in the yellow zone of the diagram (0.38, 0.53). Extending the reduction potential to more negative potentials reveals at –3.38 V, a complex ECL signal which corresponds primarily to the emissions from P1 and P2 in convolution. Significantly, the emission is stable and close to pure white in this region with a CIE coordinate of (0.35, 0.43).

## Conclusions

In this work we have described a unique ECL system comprising a single starting component capable of selectable red, green, blue or white emission depending on the applied potential. Also exceptionally, the emission colour from the complex depends on the mode of the ECL, with the co-reactant pathway producing only red emission while the annihilation pathway gives rise to various colours. An important aspect of this study is the introduction of a new facet of the use of 3D-ECL techniques, a group of methods previously only used to demonstrate selective excitation in multiplexed ECL systems. Here, we demonstrate for the first time the power of this approach for the characterisation of complex ECL mechanisms. Using this approach we have shown that the multi-coloured ECL in this system arises as a result of the formation of small traces of two highly emissive electrolysis products. At least one of the products appears to result from oxidative dissociation of a methyl group from the triazole moiety, on the basis of evidence obtained by mass spectrometry. An in-depth analysis on the photophysical, electrochemical and ECL properties of the complete family of complexes based on *fac*-tris(1-methyl-3-*n*-propyl-[1,2,4]triazolyl]iridium(iii) will be presented in a following publication.

## Experimental

### Photophysical measurements

A Nanolog (HORIBA® Jobin Yvon IBH) spectrometer has been used to record the steady-state emission spectra for a 10 micromolar solution using a 1 cm 4-sided quartz cuvette, a band pass of 5 nm, 1 nm data interval and an integration time of 0.05 s. All samples have been prepared in an air-tight cell in a N_2_-glovebox using fresh, dry and 3 times freeze–pump–thawed acetonitrile. The spectrometer contained a 450 W xenon-arc lamp as a steady-state excitation source with a 1200 g nm^–1^ grating blazed at a 330 nm excitation monochromator and the light path is directed toward a 1200 g nm^–1^ grating blazed at a 500 nm emission monochromator. A liquid-nitrogen-cooled CCD detector (Horiba Jobin Yvon, Symphony II) was used to record the steady-state emission signal. Correction factors have been used to correct the instrumental factors (light source, gratings, detectors response and *etc.*). Steady-state spectra were recorded and analysed using the FluorEssence™ (version 3.5) software package (HORIBA) and Origin 9 Pro (Origin Lab).

### Electrochemical measurements

Electrochemical experiments were performed using a μAUTOLAB type II electrochemical station potentiostat (MEP Instrument, North Ryde, NSW, Australia) with Nova 1.8 software. A conventional three-electrode cell consisting of a 3 mm diameter glassy carbon working electrode, a platinum-gauze auxiliary electrode and a silver-wire quasi reference electrode was used for all the electrochemical experiments. The working electrode was polished with 0.3 μm and then 0.05 μm alumina on a Buehler polishing pad, rinsed with Milli-Q water and then sonicated in acetone for 30 s followed by rinsing in acetonitrile and dried with a stream of N_2_. All the potentials were referenced to the ferrocenium/ferrocene (Fc^+^/Fc) (1 mM) couple as the internal standard. A stock solution of complex was prepared at a concentration of 0.5 mM in a freshly distilled, degassed acetonitrile containing 0.1 M tetra-*n*-butylammonium hexafluorophosphate (TBAPF_6_) as a supporting electrolyte. All the electrochemical experiments were performed under a nitrogen atmosphere inside a glovebox.

### Electrochemiluminescence (ECL)

For the electrochemiluminescence measurements, a photomultiplier tube (Model H7826-01 No. 62340001 Hamamatsu, Japan), biased at 500 V using a PM28B power supply (Electrons Tubes Limited, Ruislip, UK) was mounted to a custom-built electrochemical cell holder with quartz-window base. The auxiliary channel of the potentiostat was used to acquire data *via* a TA-GI-74 Ames Photonics Inc. amplifier (Model D7280). ECL spectra were obtained using a liquid-nitrogen-cooled CCD detector (Horiba Jobin Yvon, Symphony II) and a CHI 660B electrochemical station potentiostat (CH Instruments, Austin, TX, USA).

The ECL chronoamperometry experiments were carried out for 12 cycles with 0.25 s for each step potential at 0.1 V past the oxidation peak potential and at various reduction potentials. An integration time of 6 s for the CCD detector was used for each ECL spectrum.

Co-reactant ECL experiments were performed using 1 mM tri-*n*-propylamine (TPrA) in a 0.5 mM solution of complex with 0.1 M TBAPF_6_ as the supporting electrolyte. Chronoamperometry was used to pulse the potential between 0 and 0.1 V past the oxidation potential of the complex for 0.25 s. All the solutions were prepared with distilled acetonitrile dried over CaH_2_ and deoxygenated with three cycles of freeze–pump–thaw inside a light-tight N_2_ glovebox.

### 3D-ECL

#### Cyclic voltammetry

A CHI 660B potentiostat (CH Instruments, Austin, TX, USA) connected to a liquid-nitrogen-cooled CCD detector (Horiba Jobin Yvon, Symphony II) controlled using the FluorEssence™ software package (Horiba, Jobin Yvon) was used for the measurements. A triggering pulse from the CCD was sent to the potentiostat to initialize the voltammetric scan. The solution was pre-electrolyzed for 10 s at a potential of 0.49 V *versus* Fc^+^/Fc followed by a cathodic sweep from –2.46 to –3.51 V *versus* Fc^+^/Fc with a scan rate of 0.05 V s^–1^. The initial 3D-Matrix file was obtained with 21 consecutive accumulations with an integration time of 1 s from the CCD. The 3D-Matrix was then corrected to synchronize the time scale from the spectrophotometer and the potential from the potentiostat. The delay between each accumulation and the delay between sending and receiving the triggering signal were previously determined and considered in the corrected matrix. The corrected 3D-Matrix was graphed in Originlab version 9.0.

#### Chronoamperometry

A two-step potential chronoamperometry was pulsed between 0.1 V over-potential past the oxidation peak potential (0.39 V *versus* Fc^+^/Fc) and the reduction potential was varied in increments of 0.05 V from –1.81 V to –2.46 V *versus* Fc^+^/Fc. For each ECL chronoamperometry experiment 12 cycles were conducted with a pulse width of 0.25 s for each step (0.5 s for each two-step cycle, CCD integration time: 6 s). 3D-ECL plots were generated using Originlab version 9.0 to stack-plot the spectra *versus* the reduction potential.

### Mass-spectrometry

Mass spectra before and after electrolysis were recorded using a Bruker Esquire6000 mass spectrometer fitted with an Agilent electrospray (ESI†) ion source.

### Photographs

Nikon D3200 DSLR camera with a Nikon AF-S DX NIKKOR 18–55 mm f/3.5-5.6G VR lens used with ISO 400 and a 15 s exposure time for each photo.
