# Supplementary material for: Electrochemically tuneable multi-colour electrochemiluminescence using a single emitter
Source: Chem Sci. 2016 Jul 22;7(12):6974–80. doi: 10.1039/c6sc01912a (PMC5356027; doi:10.1039/c6sc01912a)
Supplement: Supplementary file 1 [file SC-007-C6SC01912A-s001.pdf]

Supporting Information for:

Electrochemically tuneable multi-colour electrochemiluminescence from a single emitter

Mohammad A. Haghighatbin,[†] Shih-Chun Lo,[‡] Paul L. Burn[‡] and Conor F. Hogan[*,†]

[†] Department of Chemistry and Physics, La Trobe Institute for Molecular Sciences, La Trobe University, Victoria, 3086, Australia

[‡] Centre for Organic Photonics & Electronics (COPE), The University of Queensland, School of Chemistry and Molecular Biosciences, Brisbane, Queensland, 4072, Australia

*To whom correspondence should be addressed. E-mail: c.hogan@latrobe.edu.au

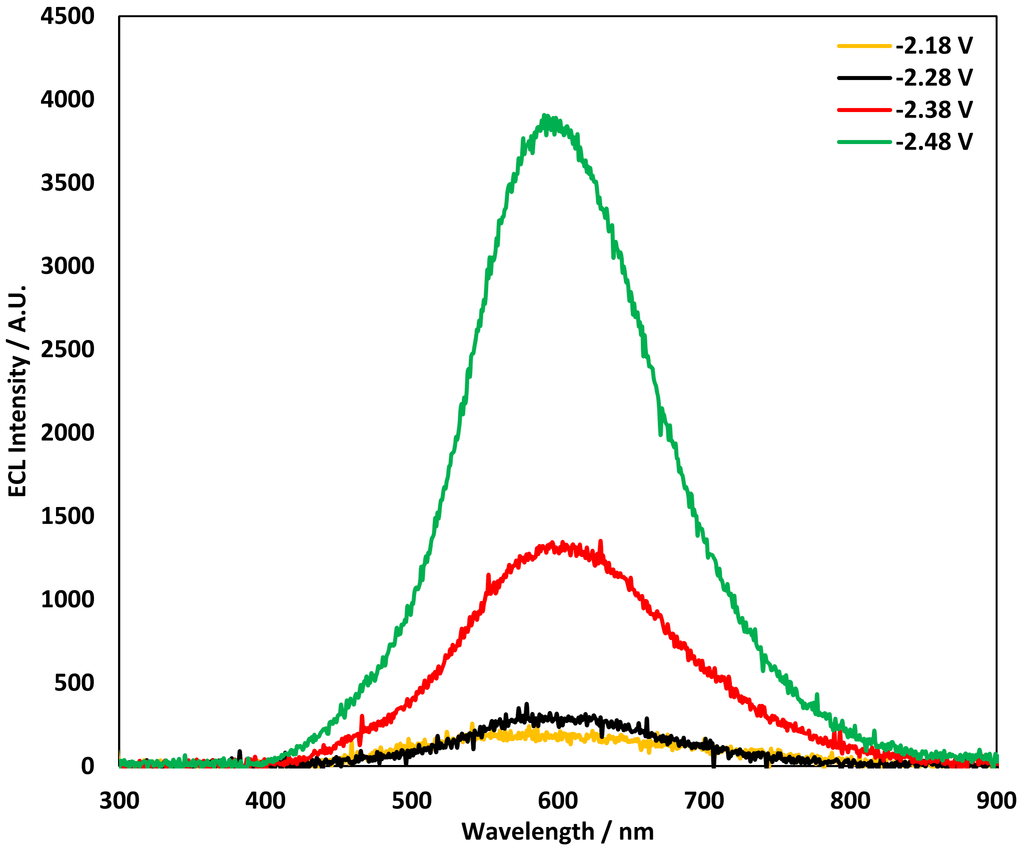

Figure S1. Red shifted annihilation ECL emission observed when cathodic potential limit was set to a value less negative than the first reduction potential of the complex. ECL spectra for (0.5 mM) solution of $Ir(mptz)_3$ and $TBAPF_6$ (0.1 M) in acetonitrile collected using a CCD detector (integration time: 6 s) and 12 cycles of two-step potential chronoamperometry with a 0.25 s pulse width between 0.49 V *versus* Fc^+/Fc and reductive potentials in the range of -2.18 V to -2.48 V *versus* Fc^+/Fc.

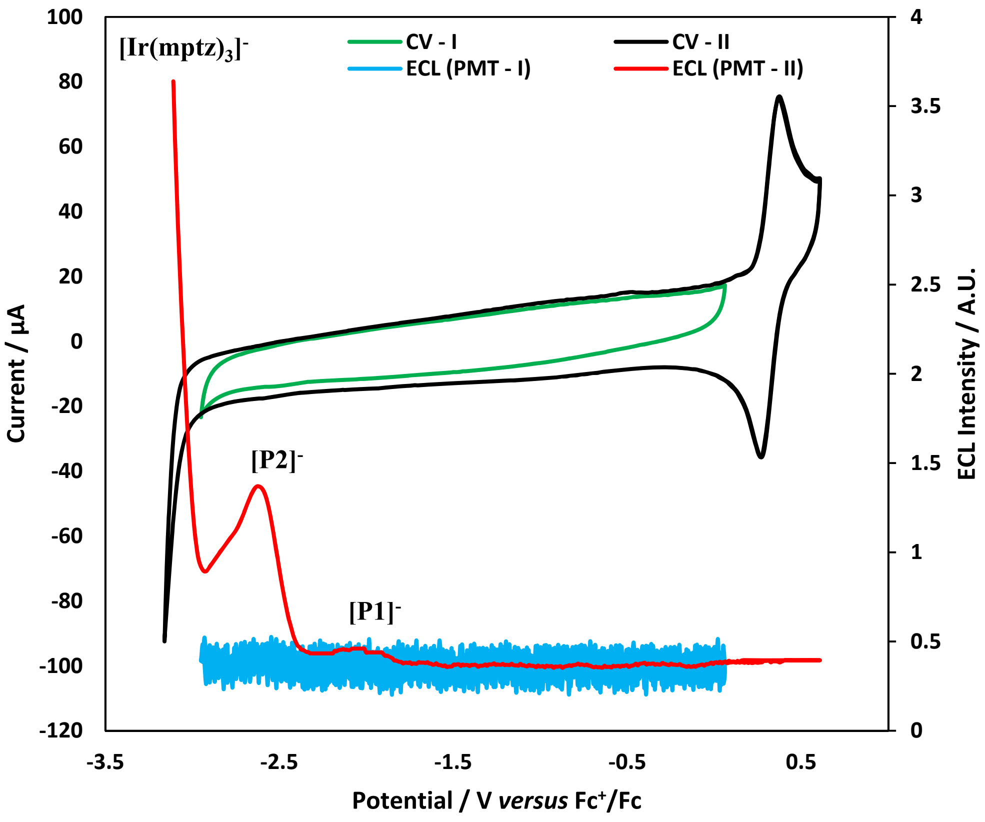

Figure S2. Cyclic voltammograms (CVs) of Ir(mptz)$_3$ (0.5 mM) in a solution of acetonitrile containing 0.1 M TBAPF$_6$ as supporting electrolyte and scan rate of 0.1 Vs^{-1} overlaid with ECL (PMT output) signal in the range of 0.6 V and -3.2 V *versus* Fc$^+$/Fc at the scan rate of 0.1 Vs^{-1}. The red and black traces are the same data as shown in Figure **2b** in the main text but with negative going scan omitted for clarity. The green and blue traces represent the control experiment, scanned at lower redox potentials (*i.e.*, 0.0 V to -3.0 V *versus* Fc$^+$/Fc).

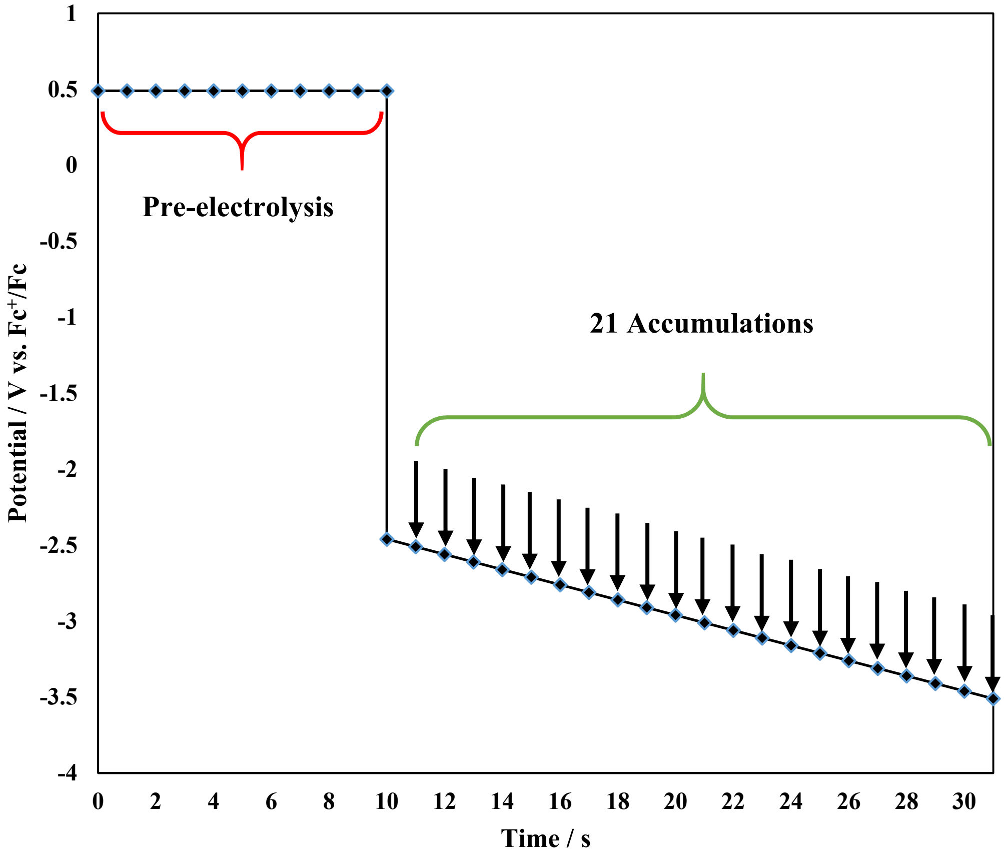

Figure S3. Excitation signal diagram for generating 3D-ECL plot (Figure **3a**). The solution pre-electrolyzed at 0.49 V *versus* Fc^+/Fc for 10 s following with a cathodic sweep from -2.46 to -3.51 V *versus* Fc^+/Fc with a scan rate of 0.05 Vs^{-1}. A CCD detector captured 21 consecutive accumulations with an integration time of 1s to form the initial 3D-matrix file.

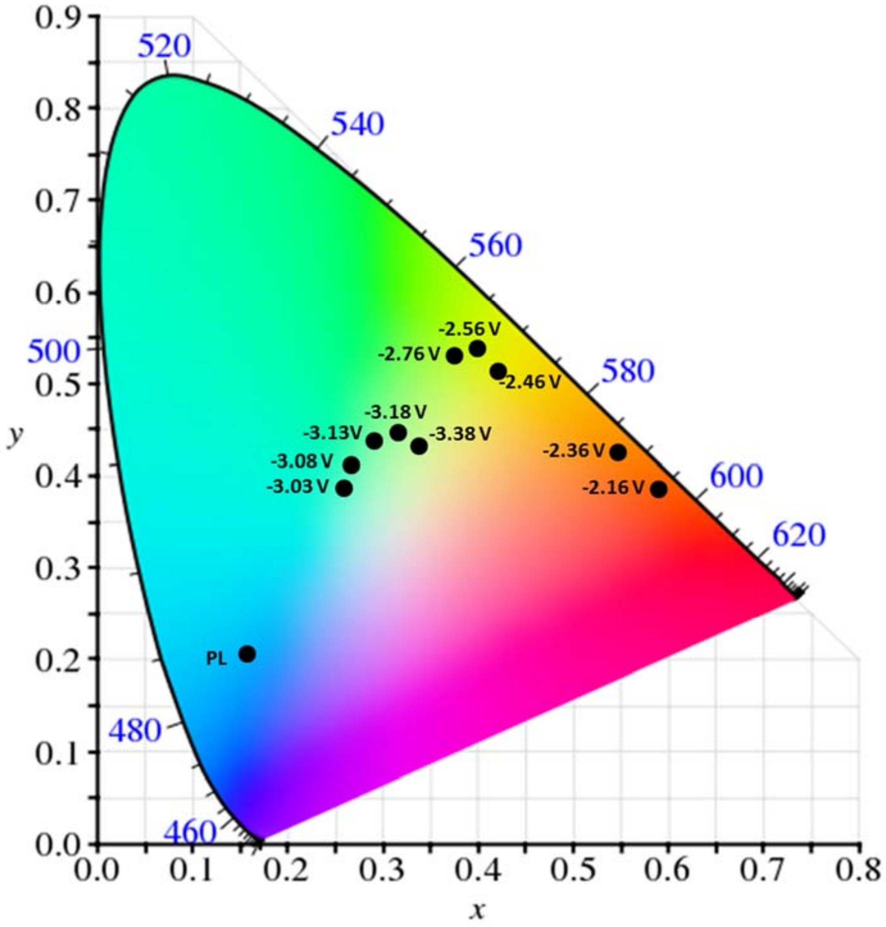

Potential / V *versus* Fc^+/Fc	CIE (x, y)
-2.16	(0.59, 0.39)
-2.36	(0.55, 0.43)
-2.46	(0.42, 0.51)
-2.56	(0.40, 0.54)
-2.76	(0.38, 0.53)
-3.03	(0.26, 0.39)
-3.08	(0.27, 0.41)
-3.13	(0.29, 0.44)
-3.18	(0.32, 0.45)
-3.38	(0.35,0.43)

Figure S4. CIE 1931 (2-degree observer) chromaticity diagram for the ECL obtained with pulsing between 0.49 V *versus* Fc^+/Fc and a range of reduction potentials (shown on the diagram and the table above).

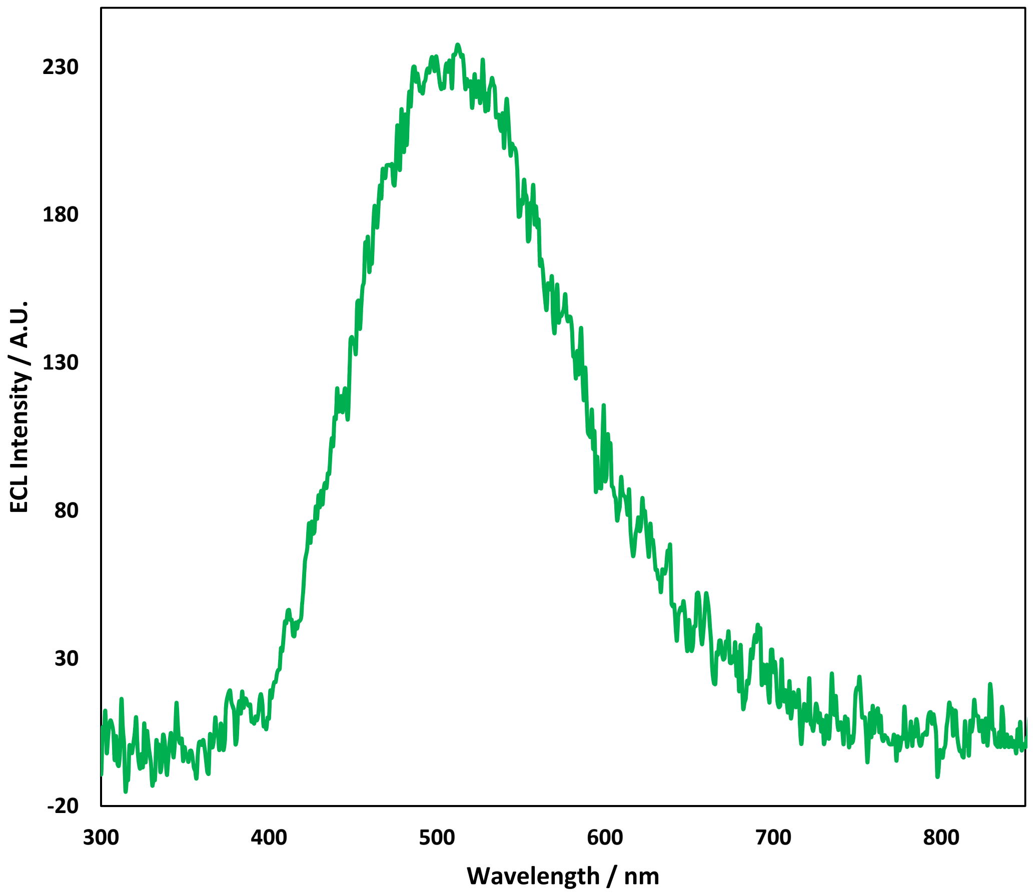

Figure S5. Near-white ECL spectrum obtained for (0.5 mM) solution of Ir(mptz)$_3$ and TBAPF$_6$ (0.1 M) in acetonitrile with pulsing the potential between 0.49 V and -3.38 V *versus* Fc$^+$/Fc.

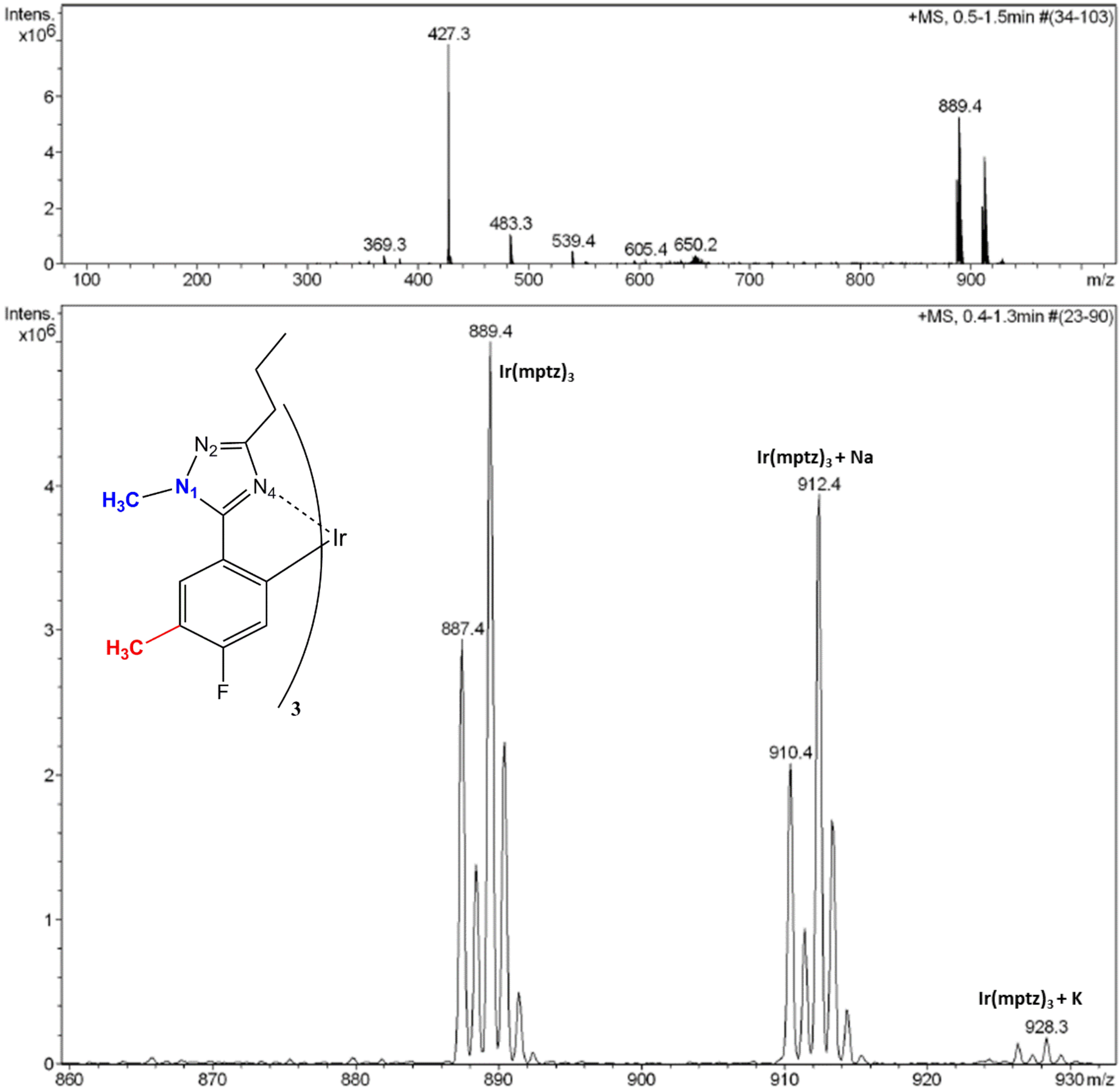

Figure S6a MS of Complex before electrochemical oxidation

Figure S6a. ESI-mass spectrum of Ir(mptz)$_3$ prior to electrochemical oxidation. The peaks at (***m/z***: 889.4, 912.4 and 928.3) are corresponded to the Ir(mptz)$_3$ complex and its sodium and potassium adducts, respectively.

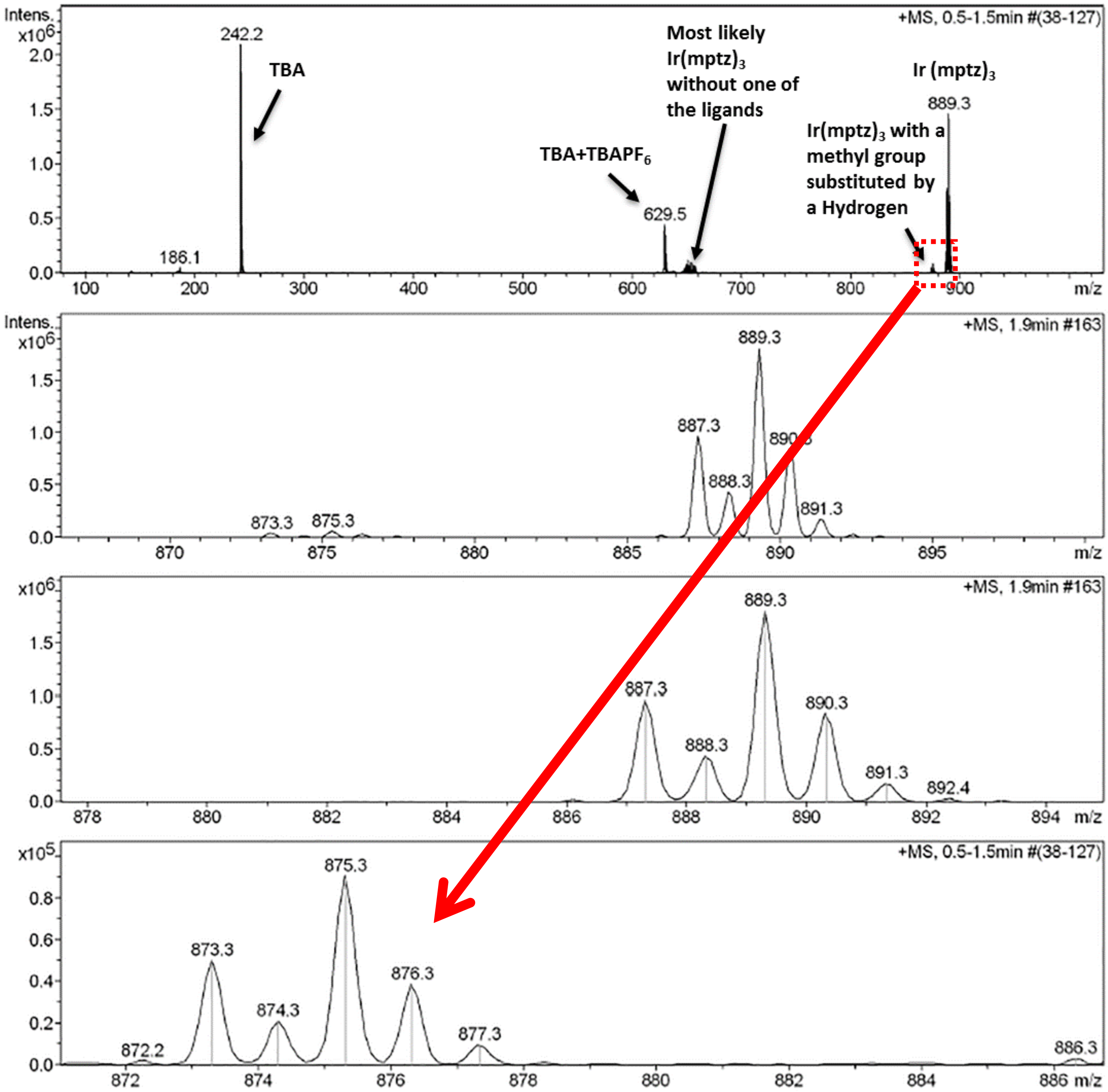

Figure S6b MS of Complex <u>after</u> electrochemical oxidation

Figure S6b. ESI-mass spectrum of Ir(mptz)$_3$ after performing bulk-electrolysis for 10 minutes 100 mV past the oxidation potential peak (**E$_{Ox}$**). The peak at (***m/z***: 875) is most likely corresponded to the product with a demethylated nitrogen (N1) on the triazole ring followed by a further protonation.
